# Retromer dependent changes in cellular homeostasis and Parkinson's disease

**DOI:** 10.1042/EBC20210023

**Published:** 2021-12-22

**Authors:** Zhe Yang, Zebin Li, Rohan D. Teasdale

**Affiliations:** School of Biomedical Sciences, Faculty of Medicine, The University of Queensland, Brisbane, Queensland, Australia

**Keywords:** cell homeostasis, Parkinsons disease, protein trafficking, retromer, sorting nexin

## Abstract

To date, mechanistic treatments targeting the initial cause of Parkinson's disease (PD) are limited due to the underlying biological cause(s) been unclear. Endosomes and their associated cellular homeostasis processes have emerged to have a significant role in the pathophysiology associated with PD. Several variants within retromer complex have been identified and characterised within familial PD patients. The retromer complex represents a key sorting platform within the endosomal system that regulates cargo sorting that maintains cellular homeostasis. In this review, we summarise the current understandings of how PD-associated retromer variants disrupt cellular trafficking and how the retromer complex can interact with other PD-associated genes to contribute to the disease progression.

## Retromer contribution to protein trafficking

Retromer protein complex, a key endosomal protein trafficking sorting platform, has been investigated in detail [1–3], establishing that not only is it involved in the retrograde cargo sorting from early endosome to the trans-Golgi network (TGN), but also from the recycling of cargo from early endosome to the cell surface. The core retromer complex consists of three subunits Vps35, Vps29 and Vps26 which functions as a scaffolding complex that engages additional accessory proteins to initiate these distinct protein trafficking pathways. In addition, two types of core retromer complex exist as defined by which Vps26 paralog they incorporate, Vps26A or Vps26B [4,5]. While some aspects of these two core retromer complexes are similar, they do for example have differences in their ability to bind cargo proteins [5].

## Cargo retrograde trafficking by retromer complex

CI-M6PR, a type I transmembrane protein, has a pivotal role in the delivery of newly synthesised lysosomal hydrolase precursor from Golgi to endosomal lumen, whereas the unoccupied CI-M6PR from the endosome can be retrieved back to the TGN for reuse. Retrograde trafficking of CI-M6PR from the endosome to the TGN can be regulated by multiple endosomal machineries including the retromer complex [6]. Previous studies had shown that core retromer of Vps35 subunit could interact with the cytoplasmic tail of CI-M6PR from yeast two-hybrid analysis, and a hydrophobic sorting motif comprising Trp-Leu-Met (WLM) within this tail is necessary for CI-M6PR retrieval [7]. The WLM sequence fits a consensus ØX[L/M/V] motif, in which Ø represents as a hydrophobic amino acid, whereas X represents as any amino acids. Several cargos recognised by retromer containing this consensus sequence, including CI-M6PR, sortilin, divalent metal transporter II (DMT1-II), Wntless, polymeric immunoglobulin receptor (pIgR) [7–9]. Among them, DMT1-II contains a short QPELYLL sequence within its cytoplasmic tail that interacts with a binding pocket between the Vps26 and SNX3 [9]. Given the conserved sorting motif between CI-M6PR and DMT1-II, the binding pocket between Vps26 and SNX3 is probably required for retromer-dependent retrograde trafficking of CI-M6PR. Other members of Vps10-related protein family are also retromer cargos retrieved from endosome to the TGN [10]. The cytoplasmic domain of sortilin contains a short sequence – FLV, which fits with a consensus ØX[L/M/V] retromer sorting motif [7] whereas SorLA instead contains a FANSHY short sequence within its cytoplasmic domain, which binds directly with retromer subunit Vps26 [11]. SorCS1, SorCS2 and SorCS3 are also cargos interacting with retromer [12,13], but the sorting motifs mediated by retromer are less clear.

The formation of one type of retrograde endosome transport carriers (ETCs) is dependent on retromer [14]. Current models indicate that the association of core retromer complex with endosomes is mediated through its concurrent interaction with Rab7 and sorting nexin 3 (SNX3), a phox homology (PX)-domain only containing protein that binds to phosphoinostide-3-phostate (PI3P) [15,16]. The formation of these ETCs is facilitated by the Wiskott–Aldrich syndrome homologue (WASH) protein complex and the detachment of these ETCs from the endosome requires the dynein/dynactin motor protein complex before delivery to the TGN [17–19]. At the level of the TGN, the ETCs interact with tethering proteins before docking and fusing with the TGN through engagement of SNARE proteins [20]. Those TGN-localised tethering proteins include golgi-associated retrograde protein (GARP), conserved oligomeric protein (COG) and golgins, such as GRIP and coiled-coil domain-containing protein 1 (GCC1 or GCC88) and Golgin subfamily A member 1(GOLGA1 or Golgin-97) [21]. Recent developments exploiting the redirection of these transient retrograde ETCs to mitochondria, using golgins engineered to be targeted to the mitochondria, enabled the direct characterisation of the sub-types of ETCs [22]. Utilisation of this approach has allowed for defining the distinct types of ETCs based on both the cargo incorporated and the protein machinery responsible for their formation [14,22,23]. For example, this approach demonstrated the retrograde trafficking of ETCs containing CI-M6PR mediated by SNX3-retromer pathway were tethered by GCC88 [14].

The interaction of the core retromer complex with the PX- BAR domains-containing SNXs has been proposed in the formation of ETCs in eukaryotes. The BAR domains of SNX-BAR proteins can stabilise and generate the high curvature of endosomal tubule structures by the regulation of membrane deformation [24]. However, the concept that the endosomal membrane tubulation capacity of the SNX-BAR proteins is essential for retromer-dependent ETC formation has been challenged. Using cryo-electron microscopy, it was demonstrated that reconstitution of core retromer trimer and SNX3 was sufficient to promote the membrane bending and the formation of tubular structures independent of SNX-BAR proteins [25]. Furthermore, the SNX-BAR proteins are not essential for the formation of retromer-dependent ETCs tethered by GCC88 but rather are required for the generation of ETCs tethered by Golgin-97 [14]. In addition, two SNX-BAR proteins, SNX5 and SNX6, can directly interact with the WLM motif of CI-M6PR to mediate CI-M6PR trafficking, therefore allowing its incorporation into ETC independent of core retromer complex [26–28]. Hence, both the core retromer complex and SNX-BAR proteins can mediate retrograde CI-M6PR trafficking via ETCs independent of each other.

## Cargo recycling by retromer complex and retriever complex

Besides retrograde transport from endosomes to the TGN, retromer can also mediate the recycling of cargo from the endosomes to the cell surface. Retromer associates with another PX domain containing protein SNX27 that contains a FERM domain at its carboxyl terminus region and a PDZ domain. SNX27's association with endosomes is mediated by the PX domain to recognise the PI3P [29], and the recognition of cargo for recycling is directed by SNX27's FERM domain that binds to a conserved NPXY/NXXY sorting motif within the cargo, including numerous receptor tyrosine kinases [30]. In addition, SNX27's PDZ domain interacts with the PDZ binding motifs (PDZbms) located at carboxyl terminus of various types of cargo [31–34]. The direct association between the PDZ domain of SNX27 and retromer Vps26 subunit promotes efficient cargo recycling by increasing the affinity of interaction of SNX27 with cargo [35]. Depletion of SNX27 often leads to reduced recycling of cargo from endosomes which results in their exposure to the endosome associated degradative pathways [36,37]. Previous proteome and bioinformatic studies have identified many proteins, including G protein coupled receptors (GPCRs), solute carrier transporters (SLCs) such as glucose transporter 1 (GLUT1), and many of other transmembrane proteins, as potential cargos for SNX27 [36,37]. Hence, SNX27-retromer has the crucial functions in the regulations of cellular homeostasis by maintaining amino acid homeostasis and signalling regulations.

Recently, there is a ‘retromer-like’ protein complex, named the retriever complex which displays structural similarity to the retromer complex was discovered [3,38,39]. The core of retriever complex is composed of Vps29, VPS35 endosomal protein-sorting factor-like (Vps35L or C16orf62) and Down syndrome critical region protein 3 (DSCR3 or Vps26C) [40]. When associated with SNX17, a FERM domain-containing SNX belonging to the same subfamily as SNX27, retriever complex can mediate recycling of cargo back to the cell surface from endosomes [40]. However, it is unclear if retriever complex also has a direct role on retrograde trafficking of cargo from endosome to the TGN.

## PD-associated retromer Vps35 variant in the etiology of Parkinson's disease

Genetic predisposition plays a critical part in the disease progression. It is estimated that ∼15 percent of people with PD have a family history of this disorder caused by genetic variants within the PD loci from Parkinson Disease 1 (PARK1) to PARK23 [41]. Particularly, PARK17 is caused by variants within retromer Vps35 gene and the functional impacts of several of these variants have been reported. Among them, Vps35 c.1858G>A (p.Asp620Asn, D620N) is the most prevalent mutation [42,43]. It has been suggested that Vps35 D620N variant acts as an autosomal dominant variant with a high but incomplete penetrance [43]. The clinical phenotypes for the patients carrying Vps35 D620N variant appear to be indistinguishable from typical idiopathic PD except for the onset of disease at an earlier age [44,45]. As our current understandings on the causes of idiopathic PD are unclear, the studying on Vps35 D620N variant would provide invaluable clues of how the idiopathic PD is initiated. In addition to Vps35 D620N variant, several variants in Vps35 have also been reported, including c.946C>T; (p.Pro316Ser, P316S) and c.1570C>T (p.Arg524Trp, R524W) [42,43]. Furthermore, several additional variants on retromer core complex, such as Vps26A c.277 A>G (p. Lys93Glu, K93E) and Vps29 c.216 A>C (p. Asn72His, N72H), have also been described associated with atypical Parkinsonism [46,47]; however, these variants appeared to be only observed in the affected individuals [47].

## Dysregulated cargo trafficking in the presence of PD-associated retromer variant

**Figure 1:**
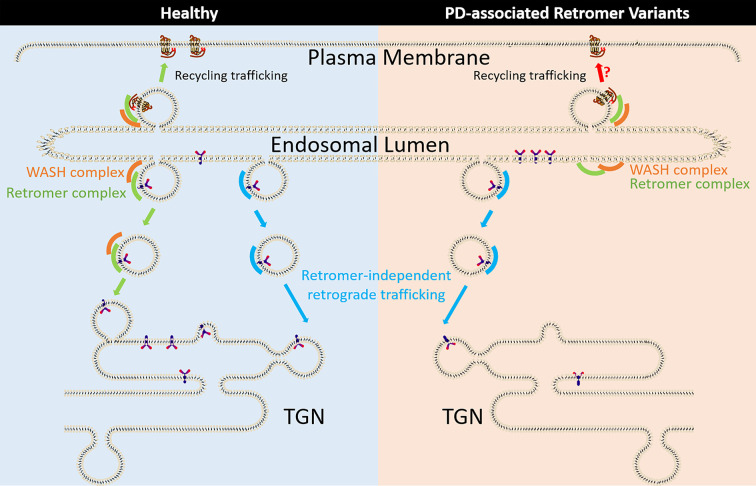
Intracellular trafficking mediated by retromer complex and PD-associated retromer variants Retrograde cargo trafficking from endosomes to the TGN is mediated through numerous pathways including retromer-dependent and independent pathways. The Retromer complex is also required for cargo recycling from the endosome back to plasma membrane. In contrast, PD-associated retromer Vps35 D620N variant is associated with changes in cargo trafficking itinerary due to the impaired binding of retromer with the WASH complex, which results in defects in formation of retrograde ETC and possibly recycling of cargo to the plasma membrane.

Recently it has been directly demonstrated the Vps35 D620N variant is deficient in the formation of retrograde ETC (Figure 1) [48]. However, the molecular mechanisms underpinning this defect in protein trafficking are not fully determined [49]. *In vitro* isothermal titration calorimetry and affinity immunoprecipitation experiments clearly demonstrate that Vps35 D620N variant has no effect on assembly of the retromer core trimer formation [50,51]. The location of Vps35 D620 in recently determined cryo-EM structures suggests that it could impact on the ability of the core retromer complex to self-assemble to form the transport vesicle coat on the membrane [25,52]. Structure modelling indicates the core retromer complex can form arch-like structures, which can be self-connected to each other through homo-dimerisation [52]. The Vps35 D620N is located near Vps35 homo-dimerisation interface, thereby potentially impacting on the affinity of Vps35 dimerisation. In addition, cryo-EM structural modelling indicates asymmetry of Vps35 within dimerised retromer complexes with one Vps35 monomer been more curved than the other [25]. This could cause the subtle changes in the orientation for these arch-like structures relative to the membrane. The presence of Vps35 D620N variant could disrupt this structural asymmetry and/or the orientation changes resulting in a decreased capacity to interact with retromer's accessory proteins and/or to assemble and stabilise the retromer core complexes as a newly formed ETC are generated (see Table 1). Indeed, biochemical evidence shows the strong inhibition of the binding between retromer with the WASH protein complex in the presence of Vps35 D620N variant [48,51,53]. The WASH protein complex contains at least five core factors, WASH1, Strumpellin and WASH-interacting protein (SWIP or WASHC4), Strumpellin, CCDC53 and Fam21, and the WASH protein complex is involved in activation of actin polymerisation to facilitate actin-dependent endosomal tubule formation and fission [19,54]. The association of retromer with the WASH complex appears to be mediated through the direct interaction between the unstructured multiple repeat elements containing the leucine-phenylalanine-acidic (LFa) motif located at Fam21's carboxyl terminus and Vps35 [55]. DnaJ homolog subfamily C member 13 (DNAJC13 or RME-8) can directly associate with SNX1 and Fam21 to mediate trafficking dynamics and cargo sorting [56,57]. In addition, Ankyrin repeat domain-containing protein 50 (ANKRD50) can regulate the cargo recycling when through association with core retromer complex, SNX27 and Fam21 [58]. Expression of the Vps35 D620N variant results in a reduced association of retromer with RME-8 or ANKRD50, indicating that the disrupted interaction between Vps35 D620N variant and Fam21 of WASH complex may represent one of the common mechanisms by which this variant leads to the mis-sorting of cargo proteins. However, the disruption of WASH complex interaction with retromer will impact on both the retrograde and recycling pathways and the details on which trafficking pathways that Vps35 D620N variant mainly affects upon in PD needs to be further resolved. Currently no WASH variants have been reported to directly associate with the PD but several variants on WASH complex subunit, such as strumpellin, have been reported to associate with other neurodegenerative diseases, including hereditary spastic paraplegia (HSP), sporadic amyotrophic lateral sclerosis and frontotemporal lobar degeneration (ALS/FTD) [59–63].

**Table 1:** Lists of retromer associated proteins and the impact of the Vps35 D620N variant

**Protein names**	**Retromer binding sites**	**Contribution to retromer function**	**Vps35 D620N association**	**References**
SNX1/SNX2 SNX5/SNX6	Vps35 and Vps29	Endosomal tubulation formation Cargo binding Cargo retrograde trafficking	Unchanged (SNX1) Not determined	[103] [104] [51]
SNX3	Vps26 and Vps35	Endosome recruitment of retromer Cargo binding Cargo retrograde trafficking	Reduced Association	[9]
SNX27	Vps26	Cargo binding Cargo recycling trafficking	Unchanged	[36,51]
Rab7	Vps26 and Vps35	Endosome recruitment of retromer	Not determined	[105,106]
TBC1D5	Vps29	Negative regulation of endosomal recruitment of retromer Autophagy regulation	Unchanged	[51,107]
VARP	Vps29	Cargo recycling trafficking	Unchanged	[51,108,109]
FAM21	Vps35 and SNX27	Cargo retrograde trafficking Cargo recycling trafficking Endosomal tubulation formation and fission	Reduced Association	[51]
ANKRD50	Vps35 and SNX27	Cargo recycling trafficking	Reduced Association	[58]
OTULIN	SNX27	Negative regulation of endosome recruitment of SNX27 Negative regulation of cargo bindings with SNX27	Not determined	[110]
PTEN	SNX27	Negative regulation of SNX27 association Negative regulation of cargo recycling trafficking	Not determined	[111]
DNAJC13 (RME8)	SNX1	Negative regulation of endosomal tubulation formation	Reduced Association	[51,56]
DLP1	Vps35	Mitochondria fission	Increased Association	[81,82]
α-synuclein (PARK1)	SNX1 and Vps29	Unknown	Not determined	[87]
Rab21	Unknown	Endosome recruitment of retromer Cargo recycling trafficking	Not determined	[112]
Rab32	Unknown	Cargo retrograde trafficking	Not determined	[113]
Rab38	Unknown	Not determined	Not determined	[112]
Rabankyrin-5	Unknown	Endosome recruitment of retromer Cargo retrograde trafficking	Not determined	[114]
VAPB	Unknown	Regulation of ER-endosome contact Regulation of vesicle budding Regulation of actin network dynamic	Not determined	[115]
MAGE-L2-TRIM27	Unknown	Cargo retrograde trafficking Cargo recycling trafficking STAT3 activation	Not determined	[116,117]
EHD1	Unknown	Stablisation of SNX1 tubules Cargo retrograde trafficking	Not determined	[118]
CASP9	Unknown	Cargo retrograde trafficking	Not determined	[119]
Bcl-xL	Unknown	Bcl-xL transport; Regulation of cell survival / death signalling	Not determined	[120]
MICAL-L1	Unknown	Bcl-xL transport; Regulation of cell survival / death signalling	Unchanged	[120]
SDCCAG3	Unknown	Cargo recycling trafficking	Reduced Association	[51,121]
FKBP15	Unknown	Cargo recycling trafficking	Reduced Association	[55]
Parkin	Unknown	Regulation of the association between retromer and the WASH complex Regulation of WASH-dependent endosomal sorting;	Unchanged	[89]
MON2	Unknown	Regulation of Wnt secretion	Not determined	[122]
PPP1R14C	Unknown	Regulation of PTH1 signalling	Not determined	[123]
GOLPH3	Unknown	mTOR signalling	Not determined	[124]
LRRK2	Unknown	Unknown	Not determined	[100]
PLA2G6	Unknown	Unknown	Not determined	[125]

Many retromer associated proteins have been reported generally via co-precipitation approaches which will capture proteins incorporated indirectly. We have highlighted the details of the retromer binding site when this interaction has been confirmed as direct using structural, ITC, yeast two hybrid or mutagenesis studies. These represent validated retromer associated proteins while we have categorised the others as unknown.

Despite numerous cargo proteins dependent on retromer being reported from several proteome studies in established cell models [28,37,64], only a handful of cargos have been identified to be mis-sorted in the presence of Vps35 D620N variant. Furthermore, the reported findings are sometimes conflicting, which may be due to the experimental systems and approaches used. One of the most established cargos mis-sorted by Vps35 D620N variant is CI-M6PR within HeLa cell line model and also human patient fibroblast cells carrying Vps35 D620N variant [48,50,51]. This is consistent with the observation of the reduced CI-M6PR protein levels in post-mortem brain tissues of idiopathic PD patients [65]. Despite the subtle defects on CI-M6PR trafficking and cathepsin processing in Vps35 D620N expressing cells, the overall lysosomal functions and degradation capacities appears to be normal [48,50]. Other independent studies did not observe any defects on CI-M6PR trafficking, instead they showed Vps35 D620N variant has the impaired capacity for autophagosome formation due to the reduced LC3B levels as well as the defects on autophagy-related protein 9A (ATG9A) trafficking [53]. ATG9A, a multi-transmembrane protein, is required for the formation autophagosome precursors, which undergoes retrograde trafficking [66]. ATG9A is primarily localised within the TGN under normal growth conditions but is re-distributed to peripheral endosomes during starvation or autophagy induction to assist in the formation of autophagosome phagophore [66]. The ATG9A trafficking is disrupted in the cells expressing Vps35 D620N variant, with ATG9A remaining localised within the TGN after autophagy induction [53]. Several retromer accessory proteins, including TBC1D5, DNAJC13 (RME8), and SNX4 – a SNX-BAR protein, have been shown to regulate ATG9A trafficking [66,67]. However, it is not known if ATG9A is a retromer cargo, hence the effect of Vps35 D620N variant on ATG9A trafficking could be indirect. Similarly, any direct impact of Vps35 D620N variant on cargo recycling still need to be clarified. For example, while it is reported that GLUT1 recycling, which is mediated through SNX27-retromer, was affected by Vps35 D620N variant, others have demonstrated unaltered GLUT1 trafficking in the presence of the Vps35 D620N variant [51,53].

A significant decrease in the protein expression of dopamine transporter 1 (DAT1), a key transporter responsible for dopamine uptake into presynaptic terminal, is consistently found in the PD patients [68], and a decrease in DAT1 also causes the loss of dopaminergic neurons in substantia nigra pars compacta in rodents [69]. Within the Vps35 D620N knockin mouse model increased dopamine release and impaired dopamine reuptake were observed by direct measurement using fast scan cyclic voltammetry in the dorsolateral striatum [70]. Consistent with these findings, the protein levels of DAT1 protein are decreased, suggesting that the Vps35 D620N variant altered DAT1 trafficking resulting in its degradation [70]. Furthermore, the very distal carboxyl terminus of DAT1 contains a short PDZbm (- LKV) and mutation of these residues reduced the recycling kinetics of DAT1, indicating that SNX27-retromer could have a role in mediating DAT1's recycling [71,72]. Vesicular monoamine transporter 2 (VMAT2) is responsible for transporting neurotransmitters from cytosol into synaptic vesicles and an increased VMAT2 protein expression was observed in Vps35 D620N knockin mouse model [70]. VMAT2 may represent a retromer cargo, as it has been shown to interact with retromer via immunoprecipitation pull-down assay [73]. VMAT2 is primarily localised within the TGN in HeLa cells while its localisation is dependent on retromer the impact of Vps35 D620N variant is unknown [73]. In addition to dopamine transporters, evidence also suggests Vps35 D620N variant may impair the recycling of excitatory AMPA-type glutamate receptors (AMPARs) to alter the excitatory synaptic transmission, as GluA1's PDZbm of AMPARs was required for binding to SNX27's PDZ domain [74–76]. Accumulated evidence indicates that Vps35 D620N variant can impact on cargo recycling, but how this variant act on this pathway needs to be further clarified. Further efforts are needed to identify the cargos, particularly in neuronal-related model systems, whose protein trafficking itineraries are impacted by the presence of the Vps35 D620N variant.

## PD-associated retromer Vps35 variant on cellular functions

Experimentally, many cellular pathways have already been implicated in the retromer-dysfunction associated with PD but their relevance is often questionable. Do they mimic the pathobiology of the disease or simply reflect an experimentally induced shift in cellular homeostasis able to cause PD associated symptoms? PD is a late onset, taking over 10–15 years to present clinically in humans and only affects a few cell types. Therefore, any predisposition to the disease caused by the genetic variant associated with familial PD can only cause subtle changes in functional capacity at the cellular level. These issues have created significant contention in the field and moving forward we need to determine the direct mechanistic molecular details that underpin the initiation of PD. α-synuclein aggregation, the major component of PD-associated Lewy bodies and associated with neuron death, represents a hallmark cellular phenotype for PD, whereas the defects on the retrograde trafficking caused by Vps35 D620N may act as one of the key drivers for the onset of the PD. Expression of Vps35 D620N variant reduces the cells ability to clear these intracellular aggregates due to the impaired endo/lysosomal functions and autophagy homeostasis [77,78]. Firstly, Vps35 D620N variant leads to the defects on CI-M6PR trafficking and lysosomal hydrolase processing [48,50,51]. Variants within glucocerebrosidase (GBA) are common risk factors for the familial PD [79]; however, a direct link between Vps35 D620N variant and GBA dysfunction has not been determined. Secondly, Vps35 D620N variant also caused the impaired autophagy homeostasis due to the defect on ATG9 trafficking [53]. Thirdly, Vps35 D620N may be also involved in the dysregulation of Lysosome-associated membrane glycoprotein 2 (LAMP2) trafficking, which is involved in chaperon mediated autophagy (CMA) [80]. Clinical features associated with the dysfunction of retromer within disease will be a consequence of the inappropriate retrograde trafficking of cargo and the resulting impact that will have on cellular homeostasis. The exact mechanisms by which Vps35 D620N variant impacts on protein trafficking itinerary of these cargos within endo/lysosomal system to induce the changes in cellular homeostasis remain to be fully determined.

Another major cellular defect caused by Vps35 D620N variant was associated with the changes on mitochondria homeostasis. Vps35 D620N variant was reported to increase its binding with dynamin-like protein 1 (DLP1), a key regulator for the mitochondria fission [81,82]. Interestingly, the carboxyl region of DLP1 contains a short FLV sequence required for retromer binding which has similarity to known retromer sorting motifs [82]. However, DLP1 is not a transmembrane protein and classified as a retromer cargo, rather, this may represent additional retromer functions beyond cargo sorting, for example, in this case, retromer and DLP1 interactions may be important for the regulation of the mitochondria and endosome contact. Regardless, this increased interaction altered the mitochondria DLP1 complexes’ turnover rate [81,82]. On the other hand, Tang et al reported that Vps35 D620N variant can impair the mitochondria functions by DLP1 independent mechanisms, by which it increases the protein level of the mitochondrial E3 ubiquitin ligase-1 (MUL1) to promote the degradation on the mitofusin-2 (MFN2), leading to the mitochondrial fragmentation [83]. The decreased enzymatic activity and respiratory defects in electron transport chain complex I and II were observed in human patient fibroblast cells deriving from Vps35 D620N variant as well as in Vps35 D620N knockin mouse model [81,82,84]. This dysregulated mitochondrion enzyme activity is also associated with the increased levels on mitochondria reactive oxygen species (ROS) and cytosolic cytochrome *c* [84]. The Vps35 D620N variant appears to alter cellular homeostasis at multiple levels by modulating the endo/lysosomal system, autophagy and mitochondrial homeostasis.

## Functional interactions between retromer and other PD-associated genes

Accumulated evidence indicates molecular interactions between retromer and other genes associated with familial PD can modify cellular homeostasis and contribute to the early-onset of disease. These include PARK1 (α-synuclein, SNCA), PARK2 (parkin), PARK6 (PTEN-induced kinase 1, PINK1) and PARK8 (leucine-repeat rich kinase 2, LRRK2). Expression of Vps35 D620N variant results in the accumulations of intracellular α-synuclein aggregates, most likely due to defects in autophagy [77,78,81,85]. In contrast, α-synuclein may also function in retrograde trafficking as a genome-wide RNA interference (RNAi) screen in HeLa identified SNCA as one of the proteins required for CI-M6PR retrograde trafficking [86]. Chemical proximity labelling using ascorbic acid peroxidase (APEX) approach and yeast two-hybrid screening have also identified a physically interaction between SNCA and retromer [87].

PINK1 (PARK2) coordinates with Parkin (PARK6) within the quality control pathway that monitors mitochondria integrity. PINK1 phosphorylates and recruits Parkin from the cytosol to damaged mitochondria, where Parkin functions as an E3 ubiquitin ligase to ubiquitinate protein substrates for proteasomal degradation which facilitates the selective remove the damaged mitochondria via mitophagy [88]. Parkin also contributes to protein trafficking through both degradative and non-degradative mechanisms. Parkin interacts with retromer to promote Vps35 polyubiquitination on multiple lysine residues, including K555, K556, K662, K663, K701 and K702 [89]. These lysine sites are located near the D620 residue and in the region that interacts with VPS29 and the WASH complex, suggesting that Vps35 polyubiquitination could be important for its function, however, the polyubiquitinated Vps35 itself is not targeted for proteasomal degradation. It remains to be determined if parkin-mediated ubiquitination on Vps35 D620N variant will influence the association of WASH complex to retromer and retromer-mediated trafficking processes.

LRRK2 (PARK8) is considering as one of the most important genetic factors contributing to PD progression. LRRK2 is a large protein containing multiple functional domains, including a kinase domain and a GTPase domain. Although LRRK2 is mainly localised to cytosol, it can be recruited to multiple intracellular compartments, including endosome, lysosome, mitochondria, ER and the TGN, from which LRRK2 mediates multiple cellular processes, such as vesicular trafficking, mitochondria homeostasis, protein synthesis and inflammatory processes [90]. Serving as a kinase, phosphoproteome studies have identified numerous substrates targeted by LRRK2 [91]. Phosphorylation by LRRK2 regulates the activity of both Rab10, a master regulator for exocytosis pathway, and Rab29 (Rab7L1), a TGN associated Rab GTPase known to regulate CI-M6PR retrograde trafficking [92–97]. In addition, the presence of Vps35 D620N can modulate LRRK2 kinase activity so that the phosphorylation of these Rab GTPases is further increased [98]. This evidence suggests retromer Vps35 D620N variant may act upstream of LRRK2. Consistent with this idea is a recent study showing LRRK2 can act as a scaffold to stabilise Golgi-associated retrograde protein (GARP) complex with the TGN-associated SNARE proteins, VAMP4 and syntaxin-6, to regulate retrograde trafficking of CI-M6PR [99]. Hence, retromer and LRRK2 may converge to act within the same retrograde protein trafficking pathway. Although it has been reported previously that retromer can bind with LRRK2 using affinity immunoprecipitation approach [100] the structural details and the regulation of this interaction remain unknown.

Overall, retromer interacts with a number of proteins encoded by other PD associated genes. These interactions are fundamentally important and contribute to the regulation of intracellular trafficking and cellular homeostasis.

## Therapeutical development to target retromer for PD and other neurodegenerative diseases

The deficiency of retromer dependent protein trafficking associated with the Vps35 D620N variant could be modulated therapeutically. Several retromer stabillisers have been developed including R55 a pharmacological chaperon that stabilises the association between Vps35 and Vps29. R55 treatment within cell models reduces the level of α-synuclein aggregation in cells expressing the Vps35 D620N variant [85]. Retromer levels in animal models can be increased by treatment with these molecules to protect against neurodegeneration in an ALS model [101] and decrease amyloid-precursor protein (APP) processing in a Alzheimer's model [102]. This raises the potential benefits of enhancement of retromer dependent protein trafficking therapeutically not just for the Vps35 D620N variant but more generally in PD.

## Summary

- Retromer complex is a key endosomal trafficking hub that mediates cargo trafficking which maintains endo-lysosomal functions and cellular homeostasis.
- Familial PD associated retromer variants have a reduced capacity to generate a single type of endosome transport carrier.
- The PD-associated retromer Vps35 D620N variant has a reduced association of the WASH complex with the retromer complex.
- Identification of the individual cargo proteins that have modified protein trafficking itineraries in PD is incomplete. This is important as any subtle changes in trafficking itinerary of individual cargo proteins has the potential to alter various associated cellular homeostatic states thereby facilitating the initiation of PD.
- As our better understanding of retromer biology continues, consideration of retromer as a therapeutic target for PD and other neurodegenerative diseases increases.

## Abbreviations

ALS: amyotrophic lateral sclerosis
AMPAR: AMPA-type glutamate receptor
ANKRD50: Ankyrin repeat domain-containing protein 50
ATG9A: autophagy-related protein 9A
CMA: chaperon mediated autophagy
DAT1: dopamine transporter 1
DLP1: dynamin-like protein 1
DMT1-II: divalent metal transporter II
DNAJC13: DnaJ homolog subfamily C member 13
FTD: frontotemporal lobar degeneration
GARP: golgi-associated retrograde protein
GBA: glucocerebrosidase
GCC1: GRIP and coiled-coil domain-containing protein 1
GLUT1: glucose transporter 1
GOLGA1: Golgin subfamily A member 1
GPCR: G protein coupled receptor
HSP: hereditary spastic paraplegia
LAMP2: Lysosome-associated membrane glycoprotein 2
LRRK2: leucine-repeat rich kinase 2
PD: Parkinson's disease
PDZbm: PDZ binding motif
PI3P: phosphoinostide-3-phostate
ROS: reactive oxygen species
SLC: solute carrier transporter
TGN: trans-Golgi network
VMAT2: Vesicular monoamine transporter 2
WASH: Wiskott–Aldrich syndrome homologue
WLM: Trp-Leu-Met
